# Editorial: Local Immune Modulation of Macrophages and Dendritic Cells - Local Matters

**DOI:** 10.3389/fimmu.2022.957104

**Published:** 2022-06-27

**Authors:** Felix Bock, Daniel Saban, Alexander Steinkasserer

**Affiliations:** ^1^Department of Experimental Ophthalmology, University of Cologne, Cologne, Germany; ^2^Department of Ophthalmology, Duke University, Durham, NC, United States; ^3^Department of Immune Modulation, University Hospital Erlangen, Erlangen, Germany

**Keywords:** microenviroment, cornea, dendritic cells, macrophages, immune modulation, transplantation

The microenvironment plays an important role in regulating immune reactions in many different diseases like ocular surface inflammation, autoimmune diseases or transplant immunology.

The eye is an example for a highly specialized microenvironment. As reviewed by Niederkorn and Taylor et al., in this Research Topic, the eye, is an immune privileged organ, which controls immune responses to injury or against pathogens very tightly to prevent irreversible damage in limited regenerative tissues, like the cornea or the retina. The maintenance of this immune privilege involves the regulation of macrophages and dendritic cells.

The ocular surface is the first frontline against environmental insults of the eye. Logeswaran et al., analyze in their study in depth the interplay between conjunctival goblet cells and dendritic cells. This study describes the micro–environmental interaction between tissue specific cells types and the immune system:goblet cells are mainly known as producers of the mucin layer of the ocular surface providing an adhesive ground for the aqueous phase of the tear film. Logeswaran et al., show that goblet cells furthermore can sense pathogens *via* the TLR 5 and subsequently locally suspend the immune regulation of adjacent dendritic cells by increasing the expression of IL6 and less activation of TGFβ.

The eye in general provides an ideal model to modulate the local immune system. Recently, it could be shown that the local application of the immune modulatory molecule sCD83, *via* preincubation of the corneal graft, induces a local as well as systemic shift of dendritic cells and macrophages towards tolerance (1).

The eye consists of macrophages from distinct ontogenies (2). Macrophages play a major role in the regulation of immune responses and therefore many contributions in this special issue focus on these cells. Hadrian et al. make a highly interesting comparison between the corneal and the skin regarding the contribution of macrophages in tissue vascularisation. Although both tissues constitute the outer barrier of our body, their properties are distinct. Both contain dendritic cells and macrophages, but whereas in the skin these cells are part of a dense vascular network, the cornea proper is free of blood and lymphatic vessels. Whereas in the skin macrophages are involved in the maintenance of the physiological blood and lymphatic vessels, corneal macrophages contribute heavily to the invasion of pathological blood and lymphatic vessels in certain inflammatory settings, but can also support the formation of lymphatic vessels only to drain corneal oedema. Therefore, the macrophages change their M1-like phenotype into M2-like phenotype with an increased expression of VEGF-C, a pro-lymphangiogenic growth factor.

The polarization of macrophages plays also an important role in other parts of the nervous system. Muhammad et al. describe in this issue how vascular macrophages contribute to formation and rupture of intracranial aneurysm (ICA). Highly interesting, also for other disciplines, is the overview of experimental M2-like polarizing agents. Our group observed recently, that a local blockade of VEGF-A in the inflamed cornea prior to transplantation increases the expression of macrophage attracting cytokines like Rantes, MCP-1, MIP-1α, MIP-1β, or GM-CSF in the corneal microenvironment (3). In the review from Muhammad et al. inhibitors such as MCP-1 are shown to reduce ICA. So, therapeutic polarization of macrophages might be a promising strategy, not only for aneurysm, but also for inflammatory or chronic diseases of the cornea, retina or skin.

Modulation of macrophage activity is not only a strategy to dampen overshooting immune responses but is also a well-known escape strategy of pathogens, e.g. *Leishmania*. Frick et al., show that not only tumors but also *Leishmania* can create an acidic microenvironment to suppress NO production and thereby the leishmanicidal activity of macrophages. Leishmaniasis can also involve the eye – the so called ocular leishmaniasis – and can cause severe damage to the ocular tissue, like corneal melting or uveitis. Other pathogens, like *Pseudomonas aeruginosa*, where recently shown to modulate the local immune response, by e.g. inducing the production of CGRP by neurons inhibiting the bactericidal activity of neutrophils (4). However, pathogens not only induce immunosuppression but also induce effective defence mechanisms. Du et al., decipher such an antibacterial mechanism induced by *Staphylococcus aureus* (*S. aureus*) in macrophages. They show how *S. aureus* induced expression of activating transcription factor 3 (ATF3) in macrophages modifies the actin-filament and thereby the motility of macrophages, resulting in an effective recruitment of antibacterial macrophages to the site of infection. In other studies ATF3 was shown to negatively regulate the activation of *NFκB* and thereby functioning as an immune regulatory factor (5). This shows how important the micro-environmental context is for shaping the local immune response.

Dendritic cells are also highly involved in local immune responses since they surveil the tissue environment. Thereby, DCs can be divided into different subtypes as described by Hongo et al. They herein described that cells from the cDC1 subtype play a role in the protection against post-ischemic acute kidney injury, a phenomenon often observed after kidney transplantation. Li et al. show that the loss of the CD1c subtype in the kidney leads to an increased Th1 response. This work demonstrates how the microenvironment is fine-tuned to avoid overshooting immune responses and on the other hand to protect from massive damage. We could recently show that the migration of dendritic cells, from the graft into lymphatic vessels, is regulated by ALCAM and how local blockade of ALCAM can inhibit this migration and consequently improve graft survival (6). Also here, fine tuning of the microenvironment could change the immune response.

In conclusion, this Research Topic demonstrates how complex local immune responses are regulated and offers a great platform to share our knowledge in respect to the immunological microenvironment on an interdisciplinary level. New therapeutic ideas as well as hypotheses will hopefully arise from the contributions of different disciplines mentioned above.

## Author Contributions

FB draft and wrote the paper. DS revised and wrote the paper. AS revised and wrote the paper. All authors contributed to the article and approved the submitted version.

## Conflict of Interest

The authors declare that the research was conducted in the absence of any commercial or financial relationships that could be construed as a potential conflict of interest.

## Publisher’s Note

All claims expressed in this article are solely those of the authors and do not necessarily represent those of their affiliated organizations, or those of the publisher, the editors and the reviewers. Any product that may be evaluated in this article, or claim that may be made by its manufacturer, is not guaranteed or endorsed by the publisher.
